# Splicing across adipocyte differentiation is highly dynamic and impacted by metabolic phenotype

**DOI:** 10.21203/rs.3.rs-3487148/v1

**Published:** 2023-10-31

**Authors:** Marcelo Nobrega, Kathryn Farris, Emil Andersen, Ida Donkin, Soetkin Versteyhe, Viggo B Kristiansen, Stephen Simpson, Romain Barres

**Affiliations:** University of Chicago; University of Chicago; University of Copenhagen; University of Copenhagen; University of Copenhagen; Sydney University; Novo Nordisk Foundation Center for Basic Metabolic Research, University of Copenhagen, Denmark

## Abstract

Adipose tissue dysfunction underlies many of the metabolic complications associated with obesity. A better understanding of the gene regulation differences present in metabolically unhealthy adipose tissue can provide insights into the mechanisms underlying adipose tissue dysfunction. Here, we used RNA-seq data collected from a differentiation time course of lean, obese, and obese with type 2 diabetes (T2D) individuals to characterize the role of alterative splicing in adipocyte differentiation and function. We found that splicing was highly dynamic across adipocyte differentiation in all three cohorts, and that the dynamics of splicing were significantly impacted by metabolic phenotype. We also found that there was very little overlap between genes that were differentially spliced in adipocyte differentiation and those that were differentially expressed, positioning alternative splicing as a largely independent gene regulatory mechanism whose impact would be missed when looking at gene expression changes alone. To assess the impact of alternative splicing across adipocyte differentiation on genetic risk for metabolic diseases, we integrated the differential splicing results generated here with genome-wide association study results for body mass index and T2D, and found that variants associated with T2D were enriched in regions that were differentially spliced in early differentiation. These findings provide insight into the role of alternative splicing in adipocyte differentiation and can serve as a resource to guide future variant-to-function studies.

## Introduction

Metabolic diseases represent a significant global health burden, with obesity and its often-associated cardiometabolic complications representing the largest contributor of this health burden (Chew et al. 2023). A key characteristic of the metabolic complications of obesity is adipose tissue dysfunction, with adipose tissue from obese individuals exhibiting abnormal phenotypes such as hypertrophy, increased inflammation, decreased differentiation capacity, and impaired insulin sensitivity (Blüther 2013; Isakson et al. 2009; Ahmed et al. 2021). These differences in obese adipose tissue function may already be present in adipocyte precursors such as preadipocytes, or may develop over the course of differentiation or in mature adipocytes (Isakson et al. 2009; Andersen et al. 2018; Longo et al. 2019). Therefore, by considering the transcriptomic and epigenomic differences between lean and obese individuals across the differentiation of preadipocytes to mature adipocytes we can gain insights into the pathophysiological mechanisms of obesity and its comorbidities.

In a previous study, we demonstrated that preadipocytes from obese subjects with or without type 2 diabetes (T2D) have distinct transcriptomic and epigenomic changes across differentiation when compared to lean individuals (Andersen et al. 2018). In all three cohorts, the transcriptome was significantly remodeled across differentiation, with thousands of differentially expressed genes between each time point comparison. Further, preadipocytes isolated from obese individuals exhibited reduced adipogenic potential that may be linked to differences in DNA methylation, suggesting that preadipocytes may be epigenetically reprogrammed *in vivo* in response to obesity and T2D. We therefore showed that gene expression and DNA methylation are significantly altered in preadipocytes isolated from obese and obese with T2D individuals when compared to lean individuals, and that there are significant transcriptomic differences between these cohorts across adipocyte differentiation that impact genes involved in important pathways such as PPAR signaling, oxidative phosphorylation, fatty acid metabolism, and insulin signaling. These results provided insights into possible mechanisms underlying adipose tissue dysfunction in obesity and T2D.

We have therefore shown that gene expression and DNA methylation changes across adipocyte differentiation are important for adipocyte biology and function. However, other forms of gene regulation may also play important roles in adipocyte function, such as alternative splicing. Specifically, alternative splicing can lead to variations in exon usage in mature mRNAs, resulting in distinct protein domains being present in the cell. This represents a regulatory strategy for modifying the cellular proteome which is independent of detectable gene expression differences measured by RNA-seq. Previous work has shown that alternative splicing in adipocytes is dynamic in response to diet-induced obesity in mice, and can be critical for key adipocyte functions such as thermogenesis (Vernia et al. 2016; Naing et al. 2023). However, relative to other modes of gene regulation, such as transcriptional regulation, the role of alternative splicing in adipocyte differentiation and function remains understudied. Splicing may play an essential role in the function of preadipocytes and adipocytes, and may underlie some differences between adipose tissue function in lean and obese individuals. In this study, we aim to systematically characterize the dynamic scope of alternative splicing during adipocyte differentiation in health and disease.

In addition to providing insights into adipocyte function and dysfunction, a better understanding of the role of splicing in adipocyte differentiation may also provide insights into the genetic underpinnings of metabolic disorders. Genome-wide association studies (GWAS) have identified hundreds of loci associated with metabolic disorders such as obesity and T2D, but the mechanisms of action for most of these genetic associations remain unclear (Yengo et al. 2018, Mahajan et al. 2018). By expanding our understanding of the scope of splicing in adipocyte differentiation and key adipocyte functions, we may be able to identify variants associated with metabolic disorders that confer risk through disrupting splicing during adipocyte differentiation, thus leading to impaired or altered adipocyte function and eventually disease. A better understanding of the splicing events that occur across adipocyte differentiation and how they are perturbed in obesity may therefore provide insights into the genetic underpinnings of metabolic disorders.

Here, we extend our previous study to consider changes in splicing across adipocyte differentiation between lean, obese, and obese with T2D humans and investigate whether any identified splicing differences may play a role in disease risk. Focusing on a subset of individuals that were deeply sequenced to identify a large number of splicing events, we identified thousands of differential splicing events across adipocyte differentiation in each cohort. We found that these splicing events largely occurred in genes that were not differentially expressed and would thus not be detectable by gene expression analyses, emphasizing the importance of considering splicing in addition to expression when measuring gene regulation changes. Finally, we considered the role these splicing events may play in the genetic risk for obesity and T2D, providing a resource to potentially help disentangle previously uncharacterized genetic associations with metabolic disease.

## Results

### Identifying splicing changes across adipocyte differentiation

To investigate the role of splicing in adipocyte differentiation, we used RNA-seq data collected from a differentiation time course of preadipocytes isolated from visceral adipose tissue of lean, obese, and obese with T2D subjects (Andersen et al. 2018, Supplementary Table 1). Isolated preadipocytes were plated and proliferated to confluence, and once they reached confluence they were induced to differentiate into adipocytes. Aliquots for RNA-seq were collected at confluence, day 3, and day 15 of differentiation. Using data collected from a subset of the original cohort (three individuals per cohort per time point, see Supplementary Table 2), we tested for differential splicing and differential expression across adipocyte differentiation in each of the three cohorts. For each cohort, we used DEXSeq (Anders et al. 2012) to test for differential splicing between confluence and day 3 and between day 3 and day 15 of differentiation, and for each time point comparison in each cohort exons with an FDR < 0.05 were considered significantly differentially spliced.

**Figure 1:**
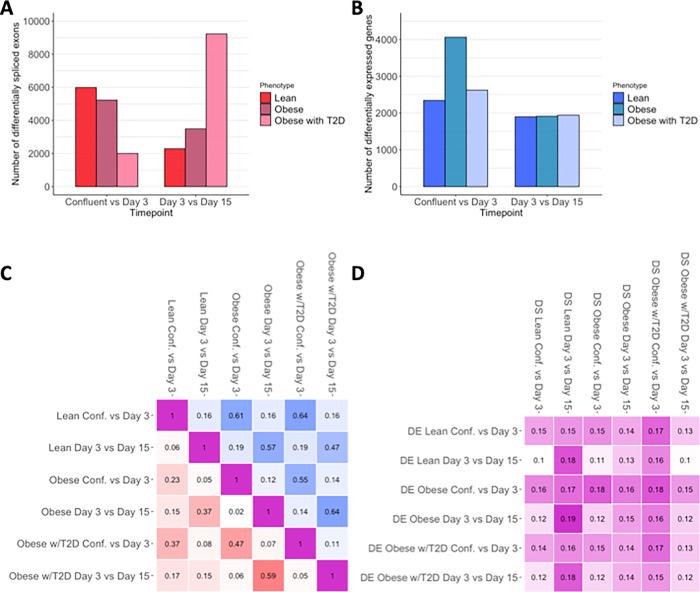
Characterizing differential splicing across adipocyte differentiation in lean, obese, and obese with T2D cohorts. A. Bar plot of the number of significantly differentially spliced exons in each cohort and each time point comparison. B. Bar plot of the number of significantly differentially expressed genes in each cohort and each time point comparison. C. Heat map of the pairwise sharing between the differential splicing sets (red) or between the differential expression sets (blue). D. Heat map of the pairwise sharing between differential expression sets and differential splicing sets. Pairwise sharing was calculated using MASH.

We found that alternative splicing is dynamic across adipocyte differentiation, with the number of differential splicing events identified at each time point comparison ranging from 1999 (in the obese with T2D cohort at confluence vs. day 3) to 9229 (in the obese with T2D cohort at day 3 vs. day 15) (Fig. 1A, Supplementary Table 3). However, the dynamics of splicing were different between the groups, with an especially pronounced difference in the obese with T2D cohort, where we saw a large number of differential splicing events during late differentiation. This pattern was the reverse of what we saw in the other two cohorts, in which there were more differential splicing events during early differentiation and fewer during late differentiation. This pattern was not apparent in the differential expression data, where all three cohorts had largely similar numbers of differentially spliced genes in later differentiation (Fig. 1B). These results demonstrate that differential splicing is pervasive and dynamic across adipocyte differentiation, that those dynamics are impacted by diseases status, and that they often are not mimicked or observable in expression data.

### Investigating patterns of gene regulation sharing between groups

After observing that there was abundant differential splicing across adipocyte differentiation in each of the three cohorts, we next investigated whether the differential splicing changes we observed were shared across the groups, and how the dynamics of splicing sharing compared to expression. Using MASH (Urbut et al. 2019), we quantified the degree of sharing between each cohort both within and across time points (Fig. 1C). We found that, overall, most of the differential splicing events identified were unique to each cohort. For example, at confluence vs. day 3 the splicing changes in the lean cohort have an estimated pairwise sharing of only 23% and 37% with the obese and obese with T2D groups respectively. The obese and obese with T2D cohorts showed higher sharing overall, with an estimated pairwise sharing of 47% at confluence vs. day 3 and 59% at day 3 vs. day 15, indicating more similarity between the splicing patterns in the two obese cohorts than between either obese cohort and the lean cohort. These results indicate that there are distinct differential splicing profiles between all three cohorts, with the lean cohort in particular showing low degrees of sharing with either of the obese cohorts.

We also investigated the degree of sharing among the differentially expressed genes, and found that they showed overall higher degrees of sharing than differential splicing (Fig. 1C). For differential expression, the pairwise sharing between any two cohorts at the same time point ranged from 47–64% as compared to 15–59% for differential splicing. For differential expression, the obese cohorts had higher degrees of sharing at later differentiation than early differentiation (64% vs 55%), in contrast to the lean cohort which had higher pairwise sharing with the obese cohorts at early differentiation as opposed to late (61% and 64% vs 57% and 47%). The amount and patterns of sharing between the cohorts therefore differ between differential splicing and differential expression, suggesting that these two forms of regulation are being affected differently by changes in phenotype.

In addition to investigating the degree of sharing within differential expression and differential splicing, we also estimated the degree of sharing between these two modes of gene regulation. We summarized the differential splicing results to the gene level by considering the most significant differential splicing change for each gene and used MASH to determine the pairwise sharing between each set of differential splicing changes and each set of differential expression changes. Overall, we found that the sharing between expression and splicing changes in this context is very low, ranging from 10–19% (Fig. 1D), indicating that analysis of gene expression alone would miss thousands of splicing events that lead to quantitative and qualitative changes in the proteome of adipocytes. These results underscore the importance of considering splicing in addition to expression when measuring the transcriptomic response of adipocytes, as splicing changes can represent a largely orthogonal form of genetic regulation that is not captured by looking at expression changes alone.

### Characterizing splicing dynamics by clustering analysis

After detecting pervasive differences in alternative splicing across differentiation that were distinct between the cohorts and largely not overlapping with gene expression changes, we next asked whether there might be distinct groups of alternative splicing events involved in specific biological processes in each cohort. We therefore sought to classify the differential splicing events we identified in each cohort into distinct response patterns. This analysis allows us to ask not just whether individual splicing events are shared or not, but whether the dynamic patterns of splicing changes overall are different between the groups. To address this question, we used fuzzy c-means clustering to cluster all the differential splicing events from each cohort across the three differentiation time points considered (confluence, day 3, and day 15). We performed a separate clustering analysis for each cohort that assigned each differentially spliced exon to one of six distinct clusters based on its dynamics across the time course (Supplementary Table 4).

Using this approach, we find that the clusters are largely shared between cohorts, although with some notable differences (Supplementary Figs. 1–3). Specifically, cluster 6 in the lean cohort, which contained exons whose usage was largely unchanged from confluence to day 3 and increased from day 3 to day 15, was not identified as one of the six strongest clusters in the obese cohort, where we instead identified the opposite - a cluster of exons whose usage was unchanged from confluence to day 3 and then decreased from day 3 to day 15. The other five clusters that were identified in the lean cohort represent dynamic patterns that were the same or very similar to clusters that were identified in the other two cohorts.

**Figure 2:**
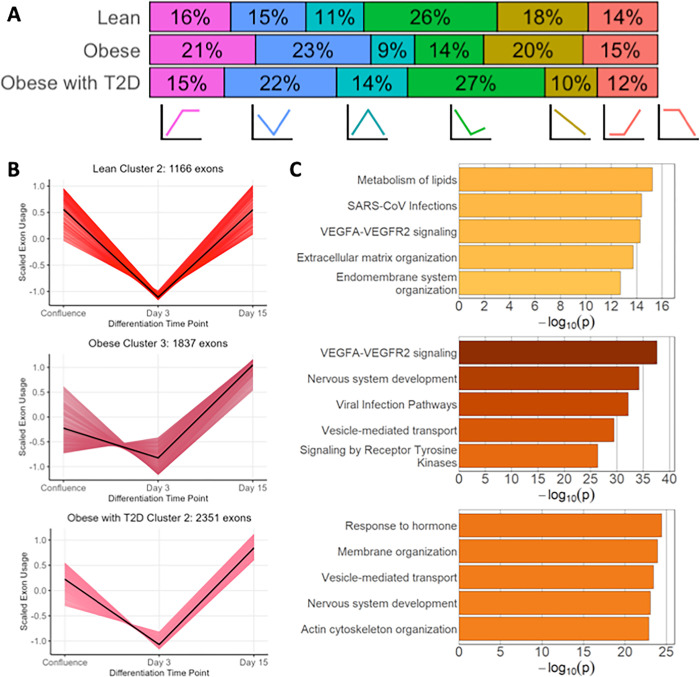
Functional enrichment differences between lean, obese, and obese with T2D cohorts within a shared splicing cluster. A. Plot showing the percent of all differentially spliced exons assigned to each of the six clusters for each cohort. Clusters are identified by a simplified representation of the cluster dynamics. B. Plots of the scaled and centered exon usage of each exon assign to lean cluster 2, obese cluster 3, and obese with T2D cluster 2. For each plot, the black line connects the cluster centroids of the cluster. C. The five most significantly enriched functional terms for each cluster, plotted against the −log_10_ of the enrichment p-value for each term.

However, although five of the six clusters were largely similar across the three cohorts, there were notable differences in the prevalence of each splicing pattern between the cohorts (Fig. 2A). When we consider the percent of total differential splicing events assigned to each cluster for each group, we find that cluster 2, which is comprised of exons whose usage decreases between confluence and day 3 and increases between day 3 and day 15, is much more prevalent in the obese and obese with T2D cohorts as compared to the lean (23% and 22% of exons compared to 15%). There were also differences in cluster prevalence between the two obese cohorts, with the obese with T2D cohort having a marked decrease in exons assigned to cluster 5 (exons whose usage decreases across all three time points) compared to both the lean and obese cohort, and the obese cohort having a marked increase in exons assigned to cluster 1 (exons whose usage increases between confluence and day 3 and is largely unchanged from day 3 to day 15) compared to both the lean and obese with T2D cohort. The three metabolic cohorts therefore exhibited distinct dynamics of alternative splicing across adipocyte differentiation.

Finally, in addition to changes in splicing dynamics across differentiation, we also observed differences in the functional enrichment of splicing events, even within a shared cluster. Lean cluster 2, which represents exons whose usage decreases between confluence and day 3 and then increases between day 3 and day 15, is also found in the obese and obese with T2D cohorts (Fig. 2B). However, functional enrichment analysis revealed key differences between the exons assigned to these three clusters (Fig. 2C). Notably, the exons assigned to lean cluster 2 are enriched for functional terms such as metabolism of lipids, VEGFA-VEGFR2 signaling, and extracellular matrix organization, indicating that this may be an important splicing dynamic for key adipocyte functions. However, the obese and obese with T2D cohorts enriched for less relevant terms such as nervous system development and viral infection pathways (although the obese cohort is also enriched for VEGFA-VEGFR2 signaling). These results demonstrate that there are both qualitative and quantitative changes in the overall dynamics of splicing between the three cohorts, and that the genes and functions being acted on by these splicing patterns have shifted in ways that may have implications for adipocyte function and development.

### Role of alternative splicing across adipocyte differentiation in GWAS for BMI and T2D

After observing that splicing is highly dynamic across adipocyte differentiation and involved in key adipocyte functions that may be perturbed in obesity, we next asked whether these splicing events might play a role in the genetic risk for metabolic diseases such as obesity and T2D. GWAS have identified hundreds of SNPs associated with metabolic disorders, and the mechanisms by which these SNPs confer risk remains largely unknown (Loos et al. 2022; Krentz et al. 2020). One possible mechanism of action for some SNPs associated with metabolic disorders is that they disrupt key splicing events during adipocyte differentiation, leading to impaired adipocyte differentiation or function. To assess whether any SNPs associated with BMI or T2D may be impacting splicing across adipocyte differentiation, we overlapped the flanking introns of differentially spliced exons in each cohort and time point with SNPs significantly associated with BMI or T2D in two large meta-analyses (Yengo et al. 2018; Mahajan et al. 2018, Supplementary Table 5). For each differential splicing analysis, we compared the number of introns overlapping at least one SNP with a null distribution generated from randomly selected exons.

**Figure 3:**
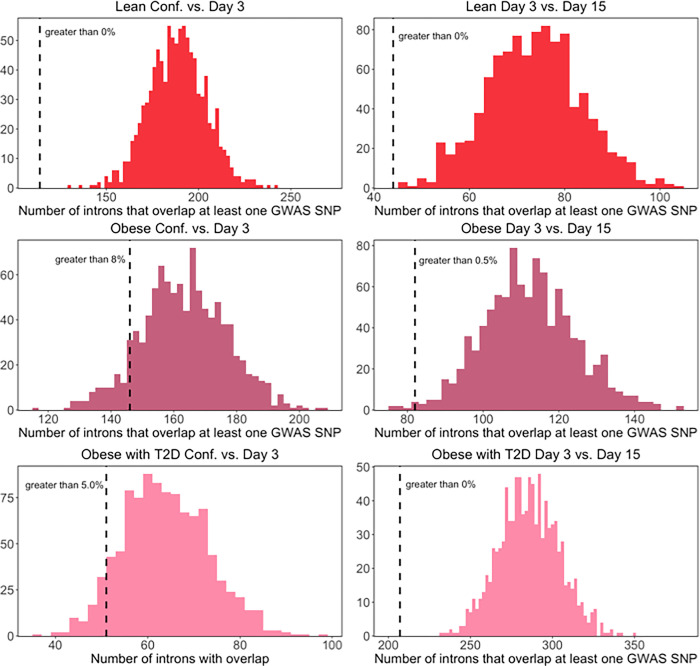
Overlap between BMI GWAS SNPs and differentially spliced exons. Histograms showing the distribution of the number of introns that overlap at least one BMI SNP across 1000 control sets of randomly selected exons. The dotted black line indicates the number of introns that overlap at least one BMI SNP in each set of differentially spliced exons.

When we performed this analysis using SNPs significantly associated with BMI, we found that there was no significant enrichment for SNPs associated with BMI in the introns of any of the differential splicing analyses tested (Fig. 3). Instead, we found that in almost every comparison there was a significant depletion of SNPs associated with BMI in the introns of differential spliced exons. This result is consistent with previous work that has found that genetic associations with BMI are strongly enriched around genes that are specifically expressed in the central nervous system (Hansen et al. 2023; Sobreira et al. 2021; Locke et al. 2015). This suggests that gene sets that are enriched for genes with specific functions in adipocytes would likely be depleted in SNPs associated with BMI. These results emphasize the complex nature of metabolic disorders and the many possible modes of action that can underlie GWAS associations.

**Figure 4:**
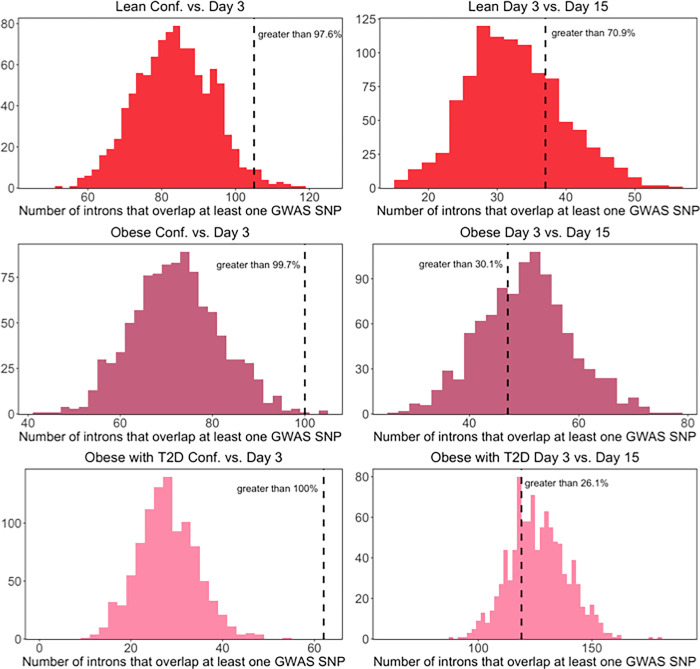
Overlap between T2D GWAS SNPs and differentially spliced exons. Histograms showing the distribution of the number of introns that overlap at least one T2D SNP across 1000 control sets of randomly selected exons. The dotted black line indicates the number of introns that overlap at least one T2D SNP in each set of differentially spliced exons.

Next, we tested for significant enrichment of SNPs associated with T2D in each of our differential splicing analyses (Fig. 4). We found that SNPs associated with T2D were significantly enriched in the flanking introns of exons that were differentially spliced between confluence and day 3 of differentiation in each of the three cohorts, but not significantly enriched in any cohort at day 3 vs. day 15 of differentiation. These results indicate that exons that are differentially spliced in the early stages of adipocyte differentiation are more likely to be functionally involved in disease risk for T2D than those that are differentially spliced in the later stages of differentiation. The differential splicing events identified here therefore provide a resource to investigate the role of differential splicing in T2D disease risk and potentially unravel the function of previously uncharacterized genetic associations with T2D. Overall, these results emphasize the importance of considering splicing as a possible disease mechanism in metabolic disorders, as well as the need to collect data across developmental time to capture possible transient associations or gene regulation changes.

## Discussion

In this study, we aimed to expand our understanding of the impact of splicing in adipocyte biology and metabolic disease. Using isolated preadipocytes collected from lean, obese, and obese with T2D individuals, we were able to generate a comprehensive catalog of alternative splicing changes across adipocyte differentiation in three different metabolic states. We identified shared and divergent splicing patterns between the three cohorts, as well as functional differences within a shared splicing pattern. Finally, we integrated our splicing results with GWAS results for BMI and T2D, generating a resource to help untangle the association between some genetic variants and disease.

Our results emphasize the importance of considering splicing in addition to expression when measuring gene regulation, as it can act as an independent regulatory mechanism from expression. We found very little overlap between differentially spliced and differentially expressed genes in every cohort and adipocyte differentiation time point assayed, with no comparison having a pairwise sharing estimate higher than 20%. This is consistent with the small overlaps found in comparisons between alternative splicing and expression in other contexts, such genes identified as differentially spliced and expressed across the cell cycle (Dominguez et al. 2016), in the aging hippocampus in mice (Stilling et al. 2014), and in a rodent model of sarcopenia (Solovyeva et al. 2021). This suggests that when considering the gene regulatory response to a perturbation or time course, it is essential to consider splicing as well as expression to capture the full spectrum of gene regulation events and changes that shape the cellular proteome, both quantitatively (measured by gene expression differences) and qualitatively (measured by splicing differences).

By expanding our understanding of the role of splicing in adipocyte differentiation and function, we can gain insight into the mechanisms of genetic risk for metabolic diseases. In the past two decades, GWAS for metabolic traits such as BMI, T2D, and WHR have provided evidence for the association of hundreds of noncoding variants with metabolic disease (Loos et al. 2022; Joslin et al. 2021; Krentz et al. 2020; Barroso et al. 2019). In an effort to elucidate the mechanism underlying these associations, previous work has used sQTL mapping to established the regulation of alternative splicing as an important linking mechanism between variant and disease (Brotman et al. 2022; The GTEx Consortium 2020; Li et al. 2016). However, some molecular QTLs have been shown to be transient over differentiation time, and would likely not be detectable in mature tissues (Strober et al. 2019). Here, we collected data from multiple time points across the differentiation of preadipocytes to adipocytes, allowing us to capture splicing events and possible variant-splice event pairs that may have been missed by looking at preadipocytes or adipocytes alone. By linking GWAS variants to splicing events across adipocyte differentiation, we can identify otherwise undetectable mechanisms possibly underlying the association of those variants with metabolic disorders. Future studies that expand the number of individuals for which we have RNA-seq time-course data could extend these results through formal sQTL mapping.

In conclusion, we have demonstrated that splicing is highly dynamic in adipocyte differentiation, is impacted by metabolic phenotype, and is acting on important adipocyte functions such as lipid metabolism and angiogenesis. We have also integrated our splicing results with genetic variants associated with BMI or T2D and generated a set of putative variant-splice event pairs that may be relevant in disease function. These results expand our understanding of the role of splicing in adipocyte differentiation and metabolic disease etiology, and can act as a resource to guide further research into the genetic underpinnings of metabolic disorders and the impact of individual splicing events on adipocyte biology.

## Methods

### Study participants

The study was approved by the Ethics Committee from the Capital Region of Denmark (reference H-1-2011-077) and informed consent was obtained from all participants. This study included a total of five lean controls, five obese subjects with T2D according to ICPC-2-DK, and four obese subjects with no history of diabetes. The participants were recruited from Surgical Gastrointestinal Department, Hvidovre Hospital, Denmark. The lean controls were subjects undergoing surgery for laparoscopic inguinal hernia repair. Individuals of both the Obese T2D and Obese groups were subjects to laparoscopic gastric bypass operation. Prior to surgery, all study participants were measured and weighted. Exclusion criteria for all three groups were: alcohol consumption of more than 14 units/week, smoking, daily intake of medicine and presence of chronic/acute diseases. Lean men with diagnosed hypercholesterolemia, hypertension and/or diabetes were excluded. Participants were fasted for at least 12 hrs and blood was drawn before undergoing anesthetics. Blood was analyzed at the Clinical Biochemistry Department, Hvidovre Hospital. Visceral adipose tissue was collected from the omental fat pat with laparoscopic surgery instruments under full narcosis during surgery.

### Isolation and culture of human preadipocytes

Isolation and culture of preadipocytes was performed as previously described (Arner et al. 2011). The adipose tissue biopsy was immediately rinsed in Phosphate-buffered saline (PBS), minced and digested by collagenase for 2½ hours in 37°C water bath shaking. Digestion was stopped by adding Dulbecco’s Modified Eagle Medium (DMEM) media supplemented with 10% Fetal Bovine Serum (FBS). The suspension was passed through a 200-μm sterile nylon filter (Spectrum Laboratories). The stromal vascular fraction (SVF) from the infranatant and the mature adipocytes from the upper fraction were washed 3 times with DMEM. The SVF was further processed through a 40-μm cell strainer and washed once in DMEM. Cells were plated at 75×106 cells/80 cm2 flask and cultured at 37°C (95% air/5% CO2) in DMEM/F12, 10% (v/v) FBS, 100 U/ml penicillin and 100 mg/ml streptomycin until 3 days prior to induction of differentiation, where FBS was removed from the media. At day 0, cells were differentiated in 5 μg/ml insulin, 10 μg/ml transferrin, 0.2 nM tri-iodothyronine (T3), 1 μM rosiglitazone, 50 μM 3-isobutyl-1-methylxanthine (IBMX) and 1 μM dexamethasone for the first 3 days. Thereafter, IBMX and dexamethasone were removed. Insulin was removed at day 12 and cells were processed at day 15.

### Nucleic acid purification and RNA sequencing

RNA and DNA were isolated with AllPrep DNA/RNA/miRNA Universal Kit (QIAGEN) according to the manufacturer’s protocols. Quality and yield were assessed by NanoDrop and Qubit dsDNA HS Assay Kit (Life Technologies). For RNA undergoing RNA-seq library preparation, RIN value was determined by Bioanalyzer instrument (Agilent Genomics), using the Agilent RNA 6000 Pico Kit. RNA-sequencing libraries were prepared using the Illumina TruSeq Stranded Total RNA with Ribo-Zero Gold protocol (Illumina) and performed as described (Nylander et al. 2016). Libraries were sequenced on a NextSeq500 instrument (Illumina) with 38-bp paired end.

### Splicing and gene expression analysis

RNA-seq reads were aligned to NCBI GRCh38 using STAR (Dobin et al. 2012). Each sample was sequenced twice, and the two sequencing runs were treated as technical replicates, with sequencing run included as covariate in both the splicing and expression analyses. Differential splicing and differential expression were calculated for each cohort (lean, obese, and obese with T2D) and for two time point comparisons (confluence vs. day 3 of differentiation and day 3 vs. day 15 of differentiation). Differential splicing was assessed at the exon level using DEXSeq (Anders et al. 2012) and for each time point comparison in each cohort exons with an FDR < 0.05 were considered significantly differentially spliced. Differential expression was assessed using limma (Ritchie et al. 2015) and for each time point comparison in each cohort genes with an FDR < 0.05 and a fold-change of at least 1.25 were considered significantly differentially expressed.

### Pairwise sharing analysis

To assess the degree of sharing between the differential splicing and expression datasets, we performed three separate analyses – one comparing all differential splicing analyses generated here, one comparing all differential expression analyses, and one comparing differential splicing and differential expression for each cohort and time point. We estimated the degree of sharing in each of these sets of analyses using mashr (Urbut et al. 2019) and a matrix of *Z* scores (for the differential splicing analysis and the joint splicing-expression analysis) or matrixes of effect sizes and standard errors (for the differential expression analysis). For the joint splicing-expression analysis, we aggregated the splicing results to the gene level by considering the most significant exon for each gene. Using mash, significant effects were considered shared if they had the same sign and were within a factor of 0.5 of each other.

### Fuzzy c-means clustering

We performed three independent clustering analyses, one for each cohort. In each, all exons that were differentially spliced in either time point comparison for that cohort were included. Differentially spliced exons were then clustered based on exon usage coefficients calculated using DEXSeq. The goal of this analysis was to capture overall patterns of expression, so exon usage coefficients were centered and scaled to account for differences in magnitude. Fuzzy c-means clustering with k = 6 was then performed on each of the three resulting datasets (for the lean, obese, and obese with T2D cohorts) using the e1071 package in R and each exon was assigned to the cluster for which it had the highest membership. Metascape (http://metascape.org, Zhou et al. 2019) was used to perform functional enrichment analysis on the gene sets associated with each cluster in each cohort.

### GWAS enrichment analysis

We first assessed the degree of overlap between each differential splicing comparison and genetic variants associated with metabolic disorder using all significant SNPs from recent GWAS of BMI (Yengo et al. 2018) and T2D (Mahajan et al. 2018). SNP coordinates were converted from hg19 to hg38 using the LiftOver tool in the UCSC Genome Browser (Haeussler et al. 2019) and intersected with the flanking introns of differentially spliced exons using bedtools (Quinlan et al. 2010). To determine if the resulting overlap represented a significant enrichment, we generated control sets of exons to compare to. For each differential splicing analysis, we generated 1000 control sets that had the same number of exons as were differentially spliced and were randomly sampled from all tested exons in that analysis. We then intersected each of those control sets with the same GWAS SNPs to generate a null distribution of the number of introns that overlap at least one GWAS SNP. Intron overlap values that were greater than 95% of the control sets were considered significantly enriched and values that were less than 5% of the control sets were considered significantly depleted.
